# mPD-APP: a mobile-enabled plant diseases diagnosis application using convolutional neural network toward the attainment of a food secure world

**DOI:** 10.3389/frai.2023.1227950

**Published:** 2023-09-25

**Authors:** Emmanuel Oluwatobi Asani, Yomi Phineas Osadeyi, Adekanmi A. Adegun, Serestina Viriri, Joyce A. Ayoola, Ebenezer Ayorinde Kolawole

**Affiliations:** ^1^Department of Computer Science, Landmark University, Omu-Aran, Nigeria; ^2^Landmark University SDG 11 (Sustainable Cities and Communities Research Group), Omu-Aran, Nigeria; ^3^School of Mathematics, Statistics and Computer Science, University of Kwazulu-Natal, Durban, South Africa; ^4^Department of Electrical and Computer Engineering, Santa Clara University, Santa Clara, CA, United States; ^5^Department of Agricultural Economics and Extension, Landmark University, Omu-Aran, Nigeria

**Keywords:** mobile-enabled, convolutional neural networks, diseases diagnosis system, SDG2, pathogens

## Abstract

The devastating effect of plant disease infestation on crop production poses a significant threat to the attainment of the United Nations' Sustainable Development Goal 2 (SDG2) of food security, especially in Sub-Saharan Africa. This has been further exacerbated by the lack of effective and accessible plant disease detection technologies. Farmers' inability to quickly and accurately diagnose plant diseases leads to crop destruction and reduced productivity. The diverse range of existing plant diseases further complicates detection for farmers without the right technologies, hindering efforts to combat food insecurity in the region. This study presents a web-based plant diagnosis application, referred to as mobile-enabled Plant Diagnosis-Application (mPD-App). First, a publicly available image dataset, containing a diverse range of plant diseases, was acquired from Kaggle for the purpose of training the detection system. The image dataset was, then, made to undergo the preprocessing stage which included processes such as image-to-array conversion, image reshaping, and data augmentation. The training phase leverages the vast computational ability of the convolutional neural network (CNN) to effectively classify image datasets. The CNN model architecture featured six convolutional layers (including the fully connected layer) with phases, such as normalization layer, rectified linear unit (RELU), max pooling layer, and dropout layer. The training process was carefully managed to prevent underfitting and overfitting of the model, ensuring accurate predictions. The mPD-App demonstrated excellent performance in diagnosing plant diseases, achieving an overall accuracy of 93.91%. The model was able to classify 14 different types of plant diseases with high precision and recall values. The ROC curve showed a promising area under the curve (AUC) value of 0.946, indicating the model's reliability in detecting diseases. The web-based mPD-App offers a valuable tool for farmers and agricultural stakeholders in Sub-Saharan Africa, to detect and diagnose plant diseases effectively and efficiently. To further improve the application's performance, ongoing efforts should focus on expanding the dataset and refining the model's architecture. Agricultural authorities and policymakers should consider promoting and integrating such technologies into existing agricultural extension services to maximize their impact and benefit the farming community.

## 1. Introduction

Attaining the United Nations Sustainable Development Goal 2 of food security, particularly in Sub-Saharan Africa, remains a pressing global challenge, for researchers and policymakers, due to the detrimental impact of plant diseases on agricultural interventions. According to Savary et al. (2019), plant diseases are responsible for the destruction of a significant portion of vital food crops, leading to substantial losses for farmers and compromised food quality. The economic consequences are also profound, with billions of dollars in agricultural investments lost to plant diseases and reduced crop yields. The lack of efficient and accessible plant disease detection technologies further exacerbates the problem, making it imperative to address this critical issue.

Plant disease detection in Sub-Saharan Africa is traditionally carried out by on-the-spot assessment of visible plant parts (Riley et al., 2002; Ma et al., 2018) due to non-existent/limited access to user-friendly advanced technologies; this has hindered accurate and efficient detection, resulting in inevitable losses in plant production. While existing research studies based on machine learning techniques have shown promises, there is a dearth of product-oriented, user-friendly, ubiquitous systems that can empower farmers in Sub-Saharan Africa, to detect plant diseases effectively and make informed decisions. This study, therefore, addresses the identified research gap by developing a novel image-processing system based on Convolutional Neural Networks (CNN) for the detection of plant diseases. The proposed system aims to provide a user-friendly and accessible solution for farmers, enabling them to identify and classify plant diseases accurately. By leveraging the power of deep learning, the system allows the computer to autonomously learn relevant features from plant images, eliminating the need for extensive human intervention. The developed web application, known as the mobile-enabled Plant Diagnosis-Application (mPD-App), represents a significant contribution to knowledge as it offers a practical tool to combat plant diseases in Sub-Saharan Africa. The robust dataset used, containing a diverse range of plant diseases, makes it suitable for crowdsourcing applications among farmers. Through this research, farmers can make timely decisions for disease management, reduce crop destruction, and enhance food security in the region, thereby aligning with the United Nations Sustainable Development Goal 2 (SDG2).

## 2. Related work

Over the past few years, there have been several significant research interventions on the development of automatic plant disease diagnosis/detection system. Some relevant studies are reviewed as follows:

Ma et al. (2018) developed a deep convolutional neural network (DCNN)-based cucumber disease detection system using leaf images. The system was used to identify four popular cucumber diseases based on their symptoms. The process included a feature-intensive segmentation of the symptoms. The dataset was augmented by flipping the images vertically and horizontally as well as angular transforms to make the dataset more robust. The authors adopted an 8:2 distribution of the training and validation sets, respectively. The DCNN was implemented by using the MatConvNet function in MATLAB. The results obtained showed that the system performed relatively well when compared with random forest, support vector machine, and AlexNet, with an accuracy of 93.4%.

Wallelign et al. (2018) developed a soybean disease recognition system based on the convolutional neural network framework. The model was trained on a 12,673 labeled sample set of soybean images taken under uncontrolled conditions. The CNN model was based on the LeNet framework and included three convolutional layers intertwined by a max pooling layer. Other processes such as segmentation and augmentation were carried out. The experiment included the distribution of the dataset as 70% training set, 10% validation set, and 20% testing set. The experimental result yielded an accuracy of 99.32%.

Picon et al. (2019) extended an earlier study by Johannes et al. (2017) on the automatic detection of various plant diseases using a deep residual neural network-based algorithm. The mobile-enabled system used 8,178 labeled sample sets against 3,637 images, according to Johannes et al. (2017). Expectedly, the additional dataset enhanced the robustness and performance of the system. The segmentation phase was carried out on symptomatic spot images of the leaf region. The deep residual neural network technique adapted was based on an earlier architecture referred to as *resnet-50*, with three convolutional layers intertwined by batch normalization and a rectifier linear unit (ReLU). The experimental result showed a promising 0.76 improvement over the study by Johannes et al. (2017). The system achieved its objective of early detection of plant diseases.

An et al. (2019) identified and classified maize drought stress using a deep convolutional neural network. The dataset was acquired using a high-end digital camera, labeled and transformed into grayscale images. In their study, they proposed the utilization of two distinct deep convolutional neural network frameworks based on ResNet50 and ResNet152. The ResNet framework mitigates image degradation and pixel explosion and improves performance. The authors adopted an 8:2 distribution of the training and validation sets, respectively. A comparative experimentation of the vis-à-vis conventional machine learning technique was carried out, and the DCNN produced a promising performance with 98.14% accuracy.

Geetharamani and Arun (2019) identified plant leaf diseases using a nine-layer deep convolutional neural network. The deep CNN model was trained using an open dataset with 39 different classes of plant leaves and background images. Six types of data augmentation methods were used as follows: image flipping, gamma correction, noise injection, principal component analysis (PCA) color augmentation, rotation, and scaling. Their model was trained using distinct training epochs, batch sizes, and dropouts, contrasted with popular transfer learning approaches. Their proposed model achieves better performance when using the validation data. After a wide simulation, the model achieved 96.46% of classification accuracy. This accuracy of the work is greater than the accuracy of traditional machine learning approaches.

In the study conducted by Durga and Anuradha (2019), SVM and ANN classifiers were implemented for the automatic detection of plant diseases. The image dataset of maize and tomato was captured using a digital camera and thereafter preprocessed to enhance the quality of the images and improve the performance of the classification algorithms. This was followed by image segmentation. The feature extraction technique deployed was the histogram of oriented gradients (HOG). The output feature set was, then, passed into the linear support vector machine (L-SVM) and artificial neural network (ANN) for classification. The L-SVM yielded an accuracy of 60%−70% for tomato and 70%−75% for maize disease detection. On the other hand, the ANN produced an accuracy of 80%−85% for tomato and 70%−75% for maize.

Chen et al. (2020), motivated by the need to improve the quality and quantity of crop production, developed an image-based plant disease identification system using a deep transfer learning technique. First, a pretrained module was implemented, essentially as a feature extractor, based on scalable feature mapping. Thereafter, a modified VGGNet which replaced ReLu with Swish activation and integrated inception modules into its layers was used to classify the image datasets of rice plant diseases. The trained model was then evaluated. The experimental evaluation showed that the prediction system had an average accuracy of 92.00%, pointing to the system's ability to effectively predict rice plant diseases.

Wang et al. (2021) developed a robust plant disease detection system to address the issue of weak detection mechanism in state-of-the-art methods. The system targeted invasive pests in tomatoes, cucumbers, and vegetables. In achieving this, the dataset's feature set was broken down into strata and recombined to allow the model to train at a finer level of detail. Additionally, the image dataset's text feature was encoded as a graph structure to make it amenable to graph convolutional neural network (GCN) used for feature extraction and training. The experimental result showed that the system performed remarkably well with an accuracy of 97.62% and precision of 92.81%.

Keceli et al. (2022) presented a robust deep learning plant detection model based on the multi-task framework, which is able to predict plant type and diseases simultaneously. They used a CNN model to train the raw image dataset and transferred latent features to a pretrained deep model. The resulting model was able to effectively and efficiently detect plant types and diseases.

Motivated by the need to improve the performance of automatic plant detection systems and make them less biased, Wang et al. (2022) developed an enhanced crop disease detection system based on convolutional neural networks. First, the crop disease image dataset was preprocessed, undergoing steps, such as resizing, normalization, denoising, and image enhancement. Then, the dataset was trained on CNN with nine convolution layers splitting the data into training and validation sets. The CNN was enhanced by integrating the Adam optimizer and other hyperparameter tuning. The experimental evaluation yielded an accuracy of 95.7%, showing that the technique can effectively detect plant diseases.

Dhiman et al. (2023) presented a precise multi-class disease detection system for fruits. The system was able to precisely detect and identify different fruit diseases based on context data fusion with faster CNN. The classification process started with the image preprocessing stage, which included normalization and dimensionality pruning of the pretrained fast CNN using polynomial decay-based sparsity. The data augmentation process was, then, undertaken based on standards that included horizontal flip, vertical flip, brightness, rescaling, shear, zca-whitening, rotation, height, and width shift. The model was, then, implemented and tuned for improved performance. The experimental result showed a high performance of a minimum of 95% on individual diseases such as scab, melanosis, greening, and black spot.

Mzoughi and Yahiaoui (2023) presented a deep learning-based segmentation for disease identification. Their objective was to leverage deep learning's computational ability to classify context-free image datasets. In this context, the dataset contains images of diseased plants without any metadata on the leaf species; in that way, the model will be robust and generalized to identify new emerging diseases. The model was trained on both context-aware and context-free datasets by combining the classification capabilities of CNN with some deep learning-based semantic segmentation techniques, namely FCN, PSPnet, and UNET. The experiment showed that, while the performance of the model on the context-free dataset was encouraging, the model performed better on the context-aware dataset.

It is noted that the reviewed studies contribute to the knowledge of plant disease diagnosis by exploring various deep learning techniques, multi-task learning, fusion strategies, and novel model architectures, to enhance the accuracy and efficiency of plant disease detection systems. While these studies produced very promising results, there is a dearth of product-oriented, user-friendly, ubiquitous system that can empower farmers in Sub-Saharan Africa, to detect plant diseases. This study presents a user-friendly and accessible solution for farmers, enabling them to identify and classify plant diseases accurately.

## 3. Materials and methods

**Figure 1:**
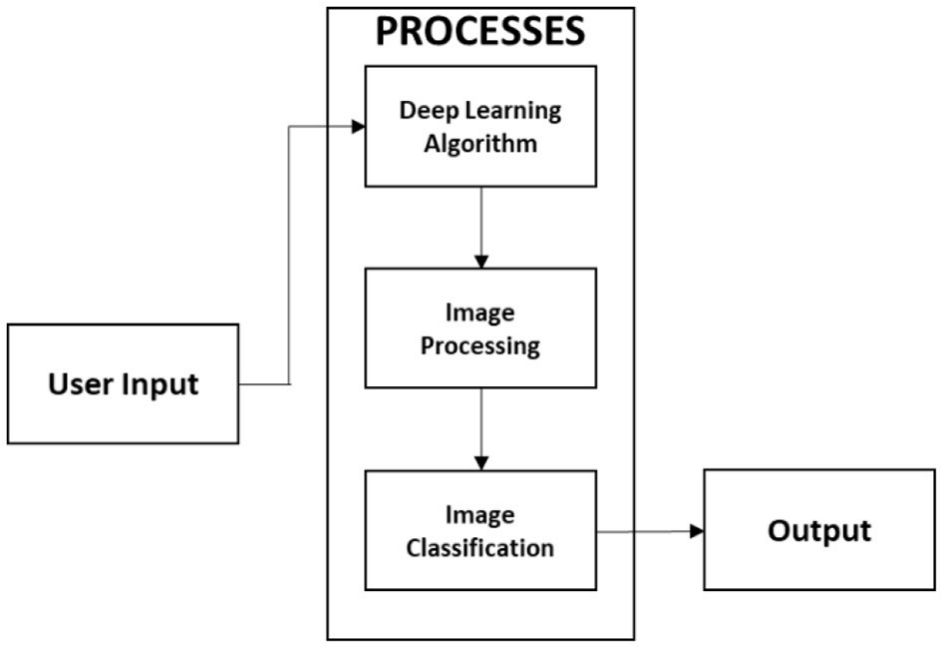
HIPO block diagram of logical processes.

In this study, we present a web-based, mobile-enabled system for the diagnosis of plant diseases, referred to as mobile-enabled Plant Diagnosis-Application (mPD-App). The system follows the traditional logical development process and is based on convolutional neural networks. Figure 1 describes the logical process of the system implementation using the hierarchical input process output (HIPO) block diagram. The system takes as input, a plant leaf image, processes the user's input, and displays the results of the diagnosis, which may either be diseased or healthy.

**Figure 2:**
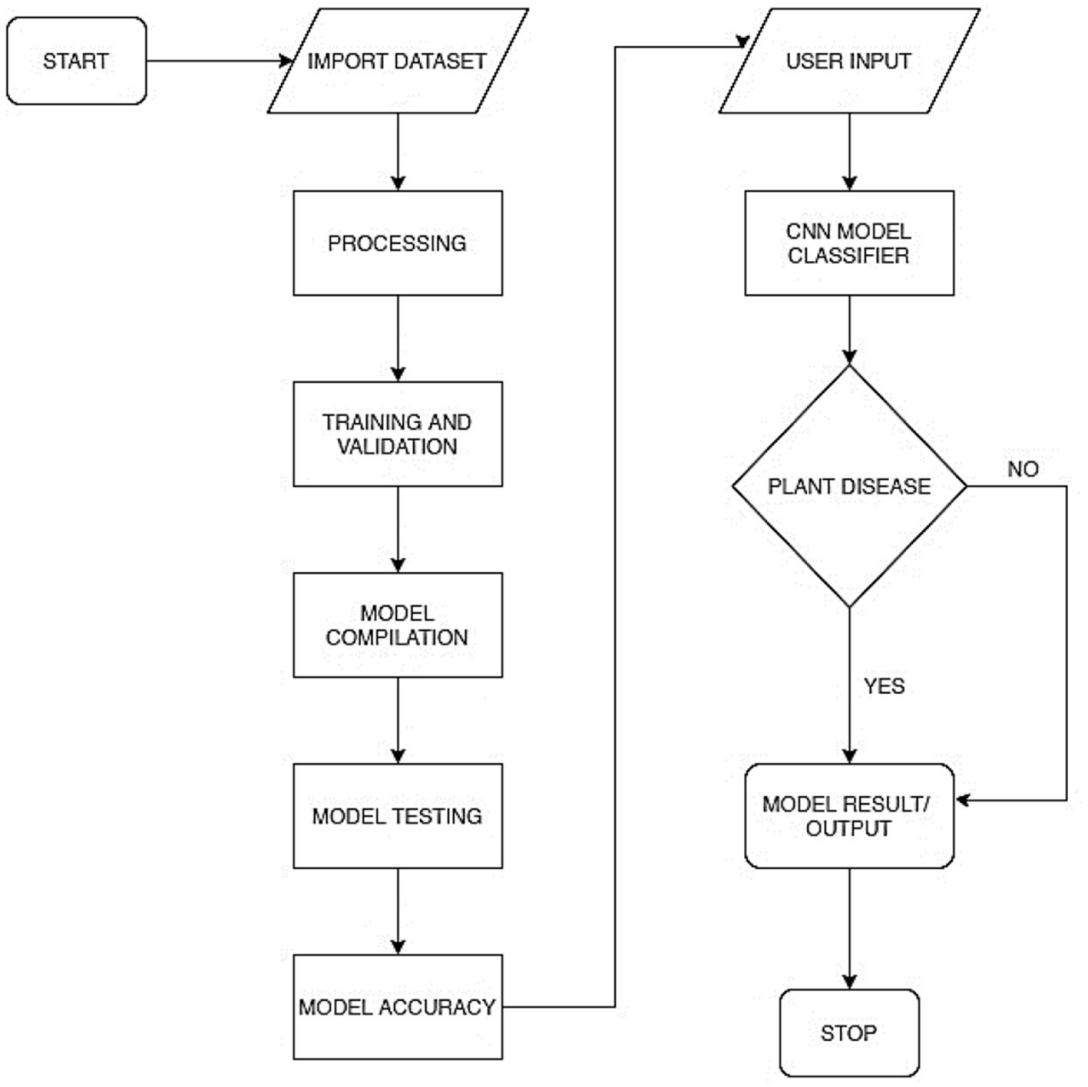
System flowchart for plant diagnosis model.

Figure 2 represents the overall system process in a flowchart. This includes the model training process and the model deployment process. The model training included processes, such as preprocessing, training and validation, model compilation, model testing, and evaluation. The model was deployed as a mobile-enabled web service. The system takes users' input, processes it *via* the embedded model, and determines whether the plant is diseased or healthy.

**Figure 3:**
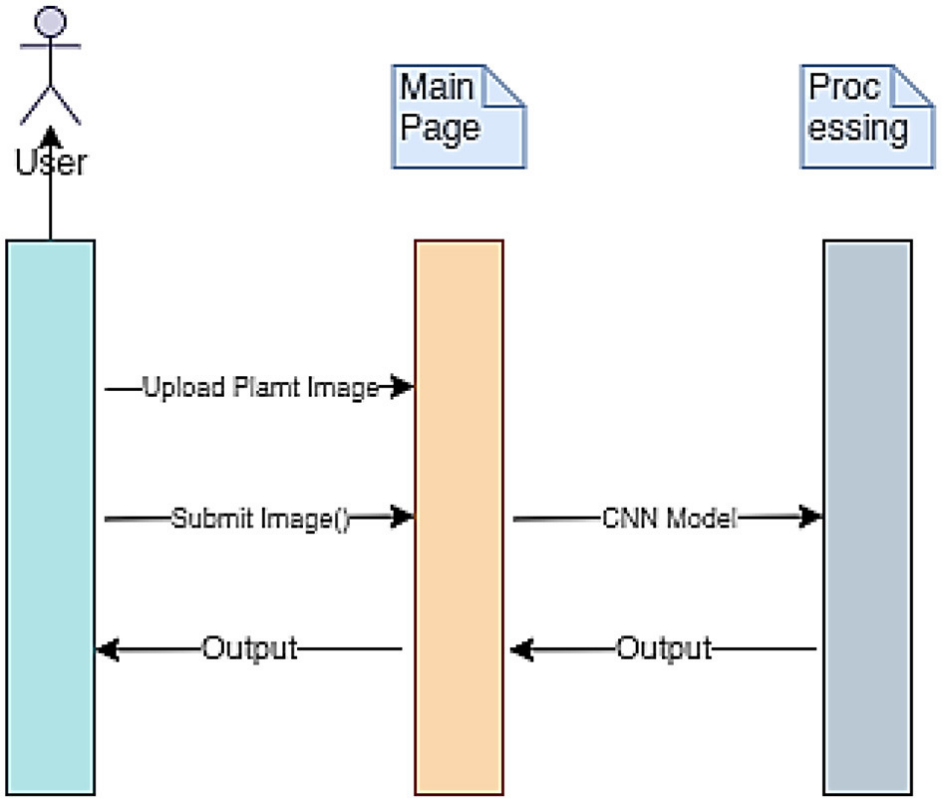
mPD-App sequence diagram.

The implementation procedure of the mPD-App is further described using the sequence diagram presented in Figure 3. The three vertical lines of mPD-App represent the three main activity layers, which include the users' layer, the system's main page, and the convolutional processing layer. The arrows represent the actions to be taken by users which in this case represent the users' input actions of uploading and submission of plant images and obtaining the output. These arrows point to the main page, which serves as the interface between the users' layer and the processing layer.

### 3.1. Data acquisition and preprocessing

**Figure 4:**
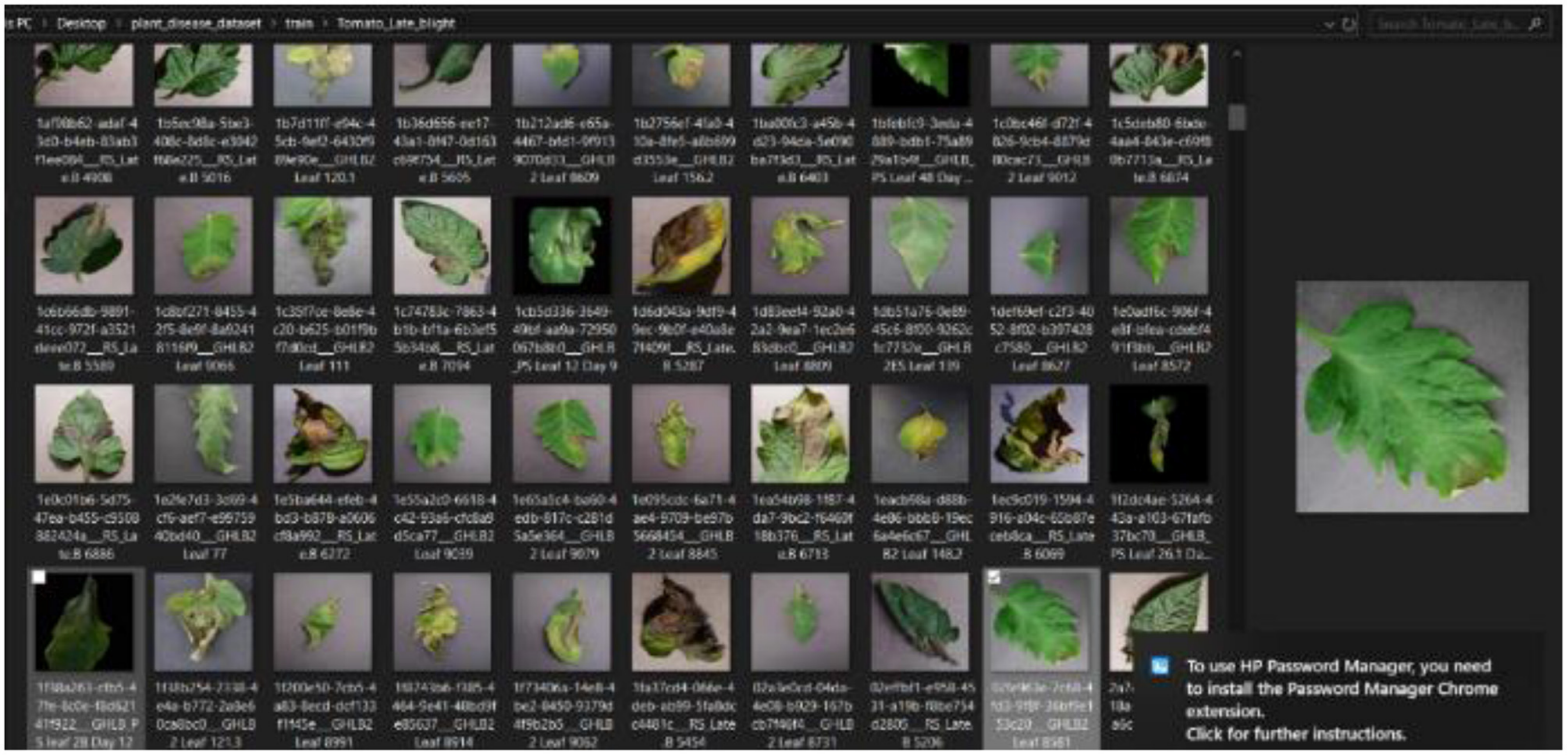
Plant disease dataset imported.

The first stage of the CNN model training and deployment involved the acquisition and retrieval of a plant disease image dataset, referred to as “*PlantVillage*,” from GitHub/Kaggle repository https://github.com/spMohanty/PlantVillage-Dataset using the search term “*plant disease dataset*.” The dataset size of 2,951 diverse range of plant leaf images (both diseased and healthy) is sufficiently significant, even as the dataset was carefully curated to ensure the inclusion of various plant diseases, such as pepper bell bacterial spot, potato early blight, potato late blight, tomato target spot, tomato mosaic virus, tomato yellow leaf curl virus, tomato bacterial spot, tomato early blight, tomato late blight, tomato leaf mold, tomato septoria leaf spot, tomato spider mites, and two-spotted spider mite, to reflect the robustness of the diagnosis system. The image dataset was, then, imported into the system. Figure 4 presents a snapshot of the plant disease dataset as imported.

In the subsequent phase, the dataset was preprocessed and invariably prepared with a format that was amenable to the CNN model. The preprocessing steps were implemented using standard functions from the Python NumPy library. The first step of the preprocessing was the image reshaping step. This was to ensure that the image sizes were uniform in dimension. Thus, the images were reshaped into 256 × 256 pixels using the “reshape” function from NumPy. The general pseudocode for image reshaping is presented in Algorithm 1. This step is crucial for compatibility with the CNN model, as it requires all input images to have the same dimensions, which, in this case, were 256 × 256 pixels.

**Algorithm 1:** Pseudocode for image reshaping.

Input: **image, image dimension**
Output: **resized image**
Get the original dimensions of the image
Calculate the scaling factors for resizing the image
**scale** = **target** **dimension**/**original** **dimension**
Initialize the new resized image
Loop through each pixel in the resized image
** Calculate the corresponding position in the original image**
Use bilinear interpolation to get the pixel value from the original image
Assign the pixel value to the corresponding position in the resized image
return resized_image

The image resizing step was followed closely by the image-to-array conversion stage. We loaded the images using the cv2 (OpenCV) library and read them iteratively from their directories for the conversion into a numerical array. During the preprocessing, the images were first converted into arrays of flat consecutive pixels using the Python function in Algorithm 2.

**Algorithm 2:** Image-to-array conversion.

def convert_image_to_array(image_dir):
try:
image = cv2.imread(image_dir)
if image is not None:
image = cv2.resize(image, default_image_size)
return img_to_array(image)
else:
return np.array([])
except Exception as e:
print (f“Error: {e}”)
return None

The final step of the preprocessing phase was normalization. The pixel values were normalized to be in the range of [0, 1] by dividing them by 255. This helped to stabilize the training process and improve convergence.

### 3.2. Model architecture

The model training was preceded by splitting the dataset into training and testing sets using the ratio 8:2. The system modeled a six-layer convolutional neural network to automatically detect plant diseases. The model included procedures, such as input layer, convolutional layer, activation layer, batch normalization layer, dense layer, and dropout. Each convolutional layer was followed by ReLU activation, MaxPooling2D, and dropout. The procedural flow is as follows:

Input **→** [Conv2D layers - Normalization - RELU - Max pooling – Dropout] **→** [Conv2D layer - Normalization - RELU] **→** [Conv2D layer - Normalization – RELU - Max pooling – Dropout] **→** [Conv2D layer - Normalization – RELU] **→** [Conv2D layers - Normalization – RELU - Max pooling – Dropout] **→** [Fully Connected Layer - Flatten - Dense layer - Normalization – RELU - Dropout - Dense layer - RELU - Dense layer]

**Figure 5:**
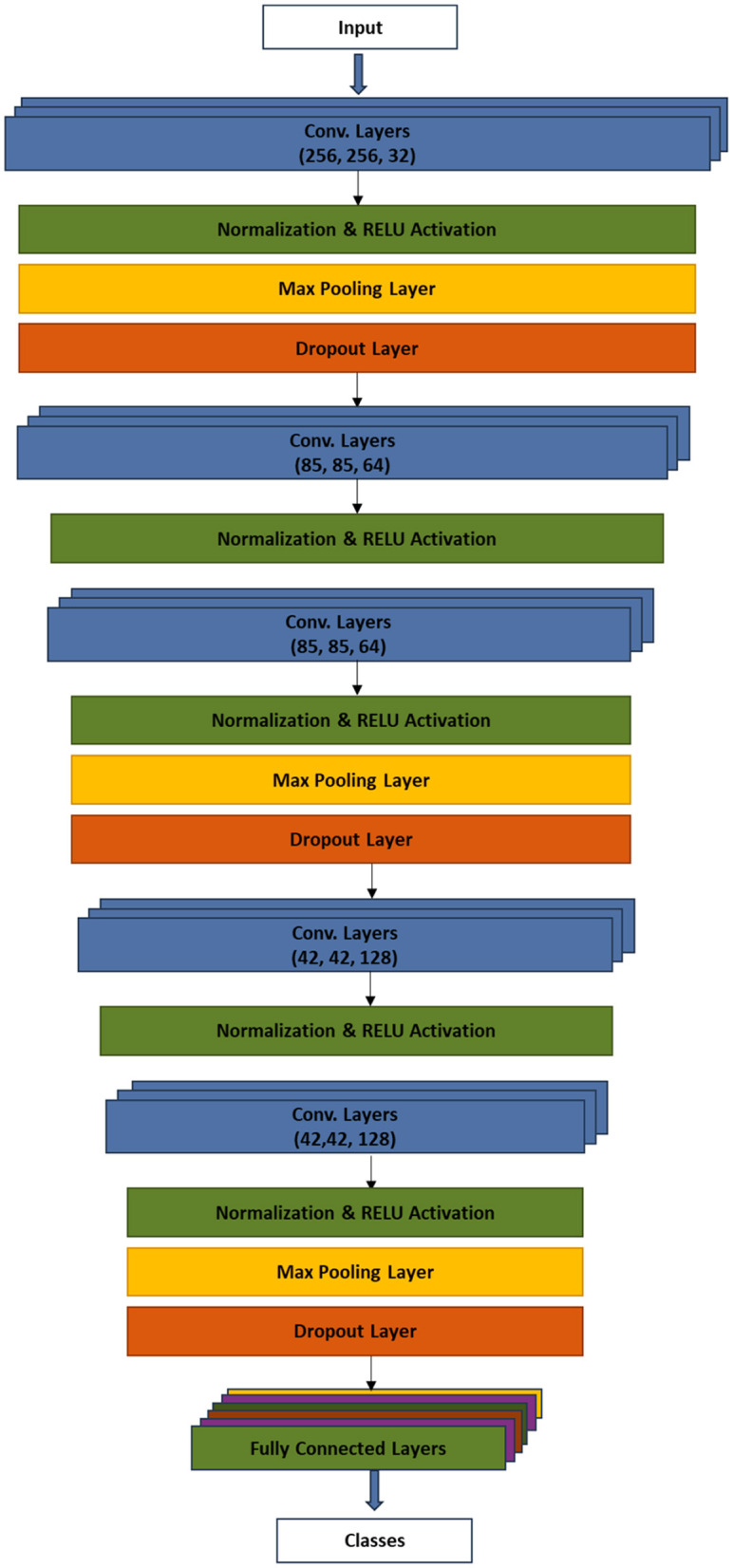
CNN plant disease detection of the model architecture.

Figure 5 presents the model architecture which depicts the major procedures of the technique.

The input layer received the preprocessed plant images, which have been reshaped to 256 × 256 pixels. The first Conv2D layer applies a set of filters to the input images to learn and extract low-level features. The batch normalization was applied to normalize images and improve the training stability. The ReLU activation function was introduced to enforce non-linearity in the network. MaxPooling2D layer performed downsampling to reduce the spatial dimensions of the feature maps. On the other hand, the dropout regularization technique was used during training to randomly set a fraction of the neurons' outputs to zero, reducing overfitting. Similar to the first Conv2D layer, the second layer extracted higher level features from the pooled feature maps of the first Conv2D layer. Again, batch normalization and ReLU activation were applied. The third convolutional layer continues to learn complex patterns, similar to the first layer, albeit with an additional max pooling and dropout. The fourth and fifth convolutional layers continue to extract intricate patterns and features from the data while adding regularization through dropout to preserve spatial information. The 3D feature maps were flattened into a 1D vector. The flattened feature set was, then, processed by the fully connected dense layer. Additional layer of batch normalization, ReLU activation, and dropout were applied. Finally, the dense layer performed the classification of plants into either diseased or healthy, using the softmax activation function.

The model was trained such that we were able to iteratively optimize its parameters (weights and biases) on the train images to minimize the loss function. The batch size was set at 32, with 15 epochs. With each iteration, the model's parameters were updated, based on the average loss across the batches. In this way, the model learns to recognize relevant patterns in the images and adjusts its parameters to make more accurate predictions. The performance of the model was evaluated on the 20% validation set to assess its generalization capabilities. The system was able to detect and diagnose 15 different plant diseases.

## 4. Results and discussion of findings

### 4.1. Web interface

**Figure 6:**
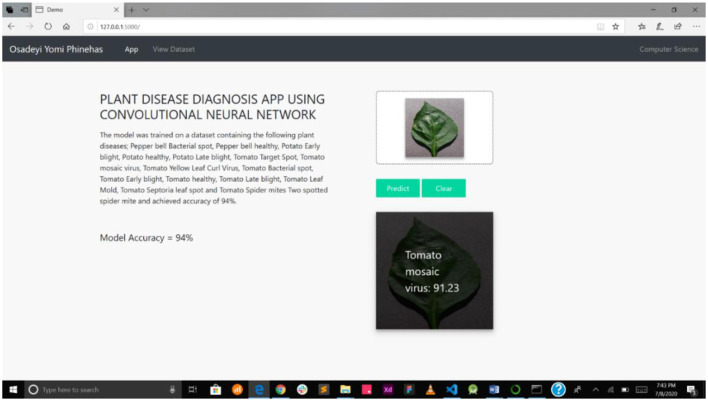
mPD-App and web interface of the plant diagnosis app.

This detection system presented in this study is a web-enabled system embedding a CNN detection model. The web interface is very simple and easy to use, showing the interaction between the users and the model. The system was implemented on a local server, as presented in Figure 6.

**Figure 7:**
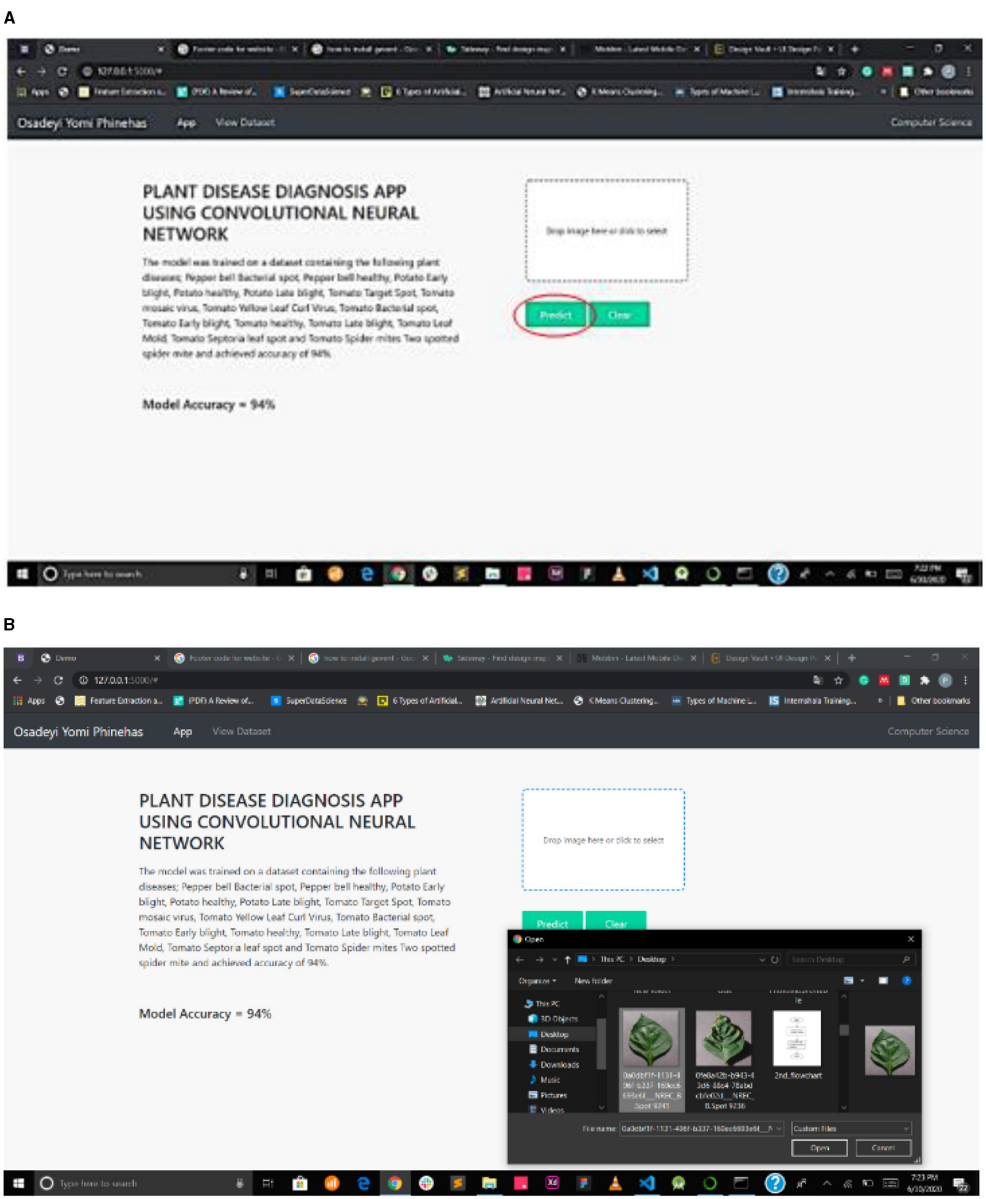
**(A)** Interface showing select field and prediction button. **(B)** mPD-App's selection process.

Figure 7A interface shows the select field and the predict button, while Figure 7B shows the mPD-App's selection process.

**Figure 8:**
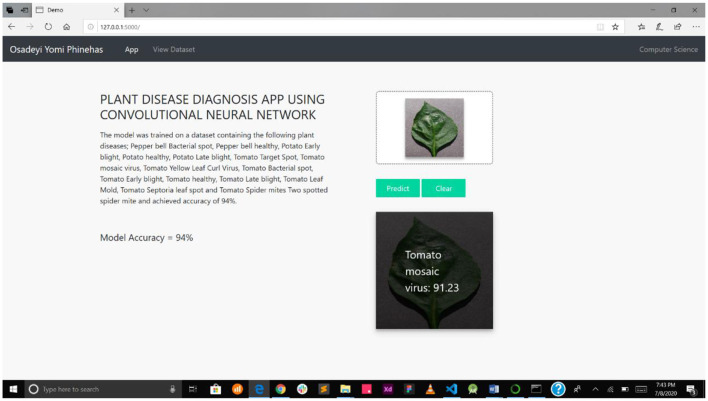
mPD-App's prediction result and the model's accuracy.

Figure 8 presents the disease diagnosis action of the system, showing both the precision and accuracy rating of the diagnosis. This will help users decide whether to trust the model's diagnosis or subject it to further scrutiny, either as part of a crowdsourcing system or experts' opinion.

### 4.2. Model's performance evaluation and discussion

**Figure 9:**
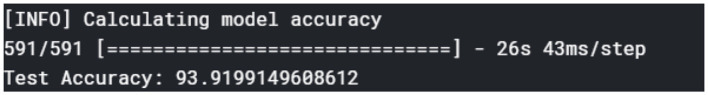
Model's overall accuracy.

In evaluating the diagnosis model, tests were performed on the validation dataset to gauge the model's performance based on metrics, such as accuracy, precision, F1-score, and recall. Figure 9 shows the overall accuracy of the model.

**Figure 10:**
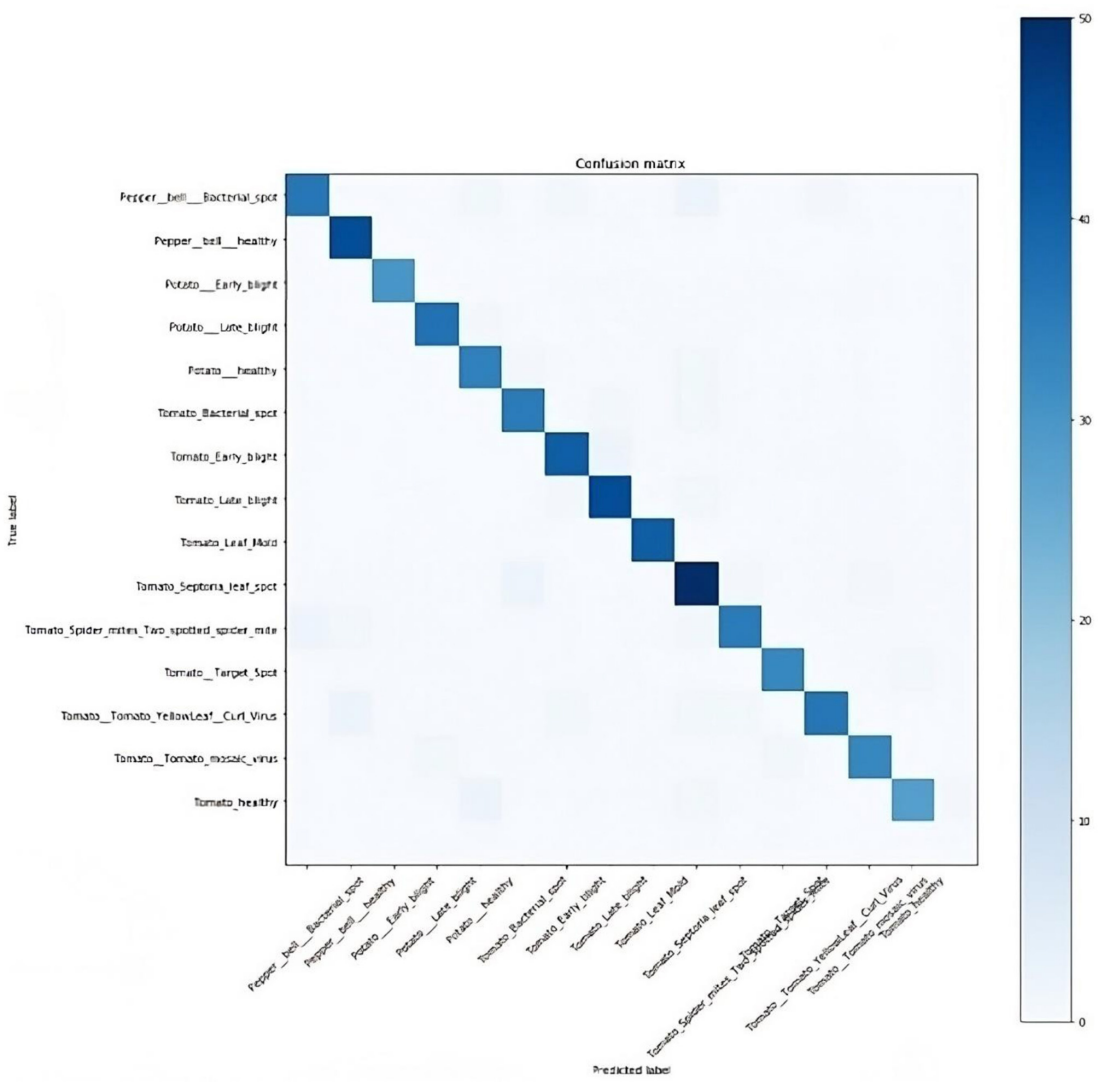
Confusion matrix.

In computing the model's performance, the true positive (*TP*), false positive (*FP*), true negative (*TN*), and false negative (*FN*) values were obtained. *TP* depicts the number of healthy plants classified correctly, *FP* represents the number of diseased plants classified as healthy, *TN* represents the number of diseased plants correctly classified as diseased, while *FN* is the number of healthy plants classified as diseased. Based on this, the confusion matrix, as presented in Figure 10, was plotted to show the performance of the model, that is, if the diagnoses matched the actual data.

Accuracy is the ratio of the total number of instances correctly diagnosed to the total amount of data in the analysis. The accuracy of the plant disease diagnosis was computed using the formula in Equation (1).


(1) $$\begin{array}{l} Accuracy\;=\frac{\left(TP+TN\right)}{\left(TP+TN+FP+FN\right)} \end{array}$$

The plant disease diagnosis model achieved an overall accuracy of 94%, demonstrating its ability to effectively and correctly detect plant diseases. Its performance in diagnosing the varying plant diseases was also promising, as presented in Table 1.

**Table 1:** Model evaluation for individual class label.

**S/N**	**Class label**	**Precision**	**Recall**	**F1-score**
1	Pepper bell bacteria spot	0.95	0.88	0.91
2	Healthy pepper bell	0.94	1.00	0.97
3	Potato early blight	1.00	1.00	1.00
4	Potato late blight	0.97	0.97	0.97
5	Healthy potato	0.89	0.94	0.92
6	Tomato bacteria spot	0.92	0.95	0.93
7	Tomato early blight	0.93	0.95	0.94
8	Tomato late blight	0.94	0.96	0.95
9	Tomato leaf mold	1.00	1.00	1.00
10	Tomato septoria leaf spot	0.86	0.93	0.89
11	Tomato spider mite	0.95	0.90	0.92
12	Tomato target spot	0.97	0.97	0.97
13	Tomato yellow leaf curl virus	0.97	0.88	0.92
14	Tomato mosaic virus	0.97	0.94	0.96
15	Healthy tomato	0.97	0.88	0.92
Accuracy				0.94
Weighted average		0.95	0.94	0.94

The precision shows the ratio of correctly predicted positive observation for each class. The precision of the model was computed using the formula in Equation (2). The mPD-App has an overall precision rate of 95%. This indicates that the model was very effective in correctly diagnosing plant diseases.


(2) $$\begin{array}{l} Precision\;=\frac{\left(TP\right)}{\left(TP+FP\right)} \end{array}$$

The recall, also called sensitivity, was used to measure the proportion of correct positive values among those that are actually positive. The mPD-App has an overall recall rate of 94%. The model was able to detect to a very high degree that plant diseases were actually plant diseases.


(3) $$\begin{array}{l} Recall\;=\frac{\left(TP\right)}{\left(TP+FN\right)} \end{array}$$

The F1-score of the model was computed by obtaining the model's weighted average of both precision and recall, as shown in Equation (4). The mPD-App has an overall F1-score rate of 94%.


(4) $$\begin{array}{l} F1\;=\;\frac{2\;*\;precision\;*\;recall}{precision\;+\;recall\;} \end{array}$$

**Figure 11:**
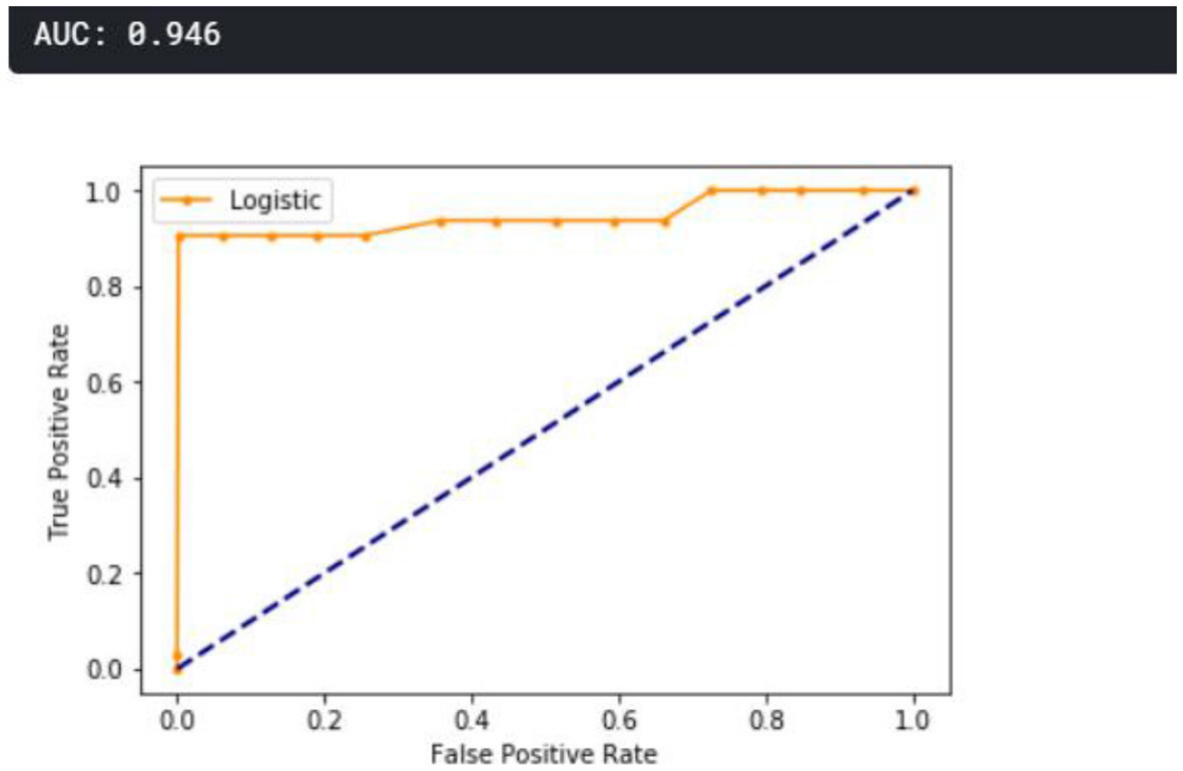
ROC curve.

A receiver operating characteristic curve (ROC) curve is plotted and presented in Figure 11. The true positive is plotted against the false positive, as shown below. The area under the curve (AUC) shows that the cumulative performance across all classification thresholds gave a result of 0.946, which implies that the developed model is good.

The model was compared with some state-of-the-art plant disease detection systems. We note that most efforts in the literature focused on the development of models, without necessarily deploying them as web-based, user-friendly products. Our choice of comparative studies was informed by their deployment, method, and similarity in dataset and disease detection. Ma et al. (2018) were chosen based on a deep convolutional neural network. Their system achieved an accuracy of 93.4%. Picon et al. (2019) used a deep residual neural network-based algorithm to develop a mobile-enabled system. It is, therefore, similar to our system in terms of method and deployment, hence its choice. Their system achieved an accuracy of 87%. According to Durga and Anuradha (2019), the SVM and ANN models were used to detect maize and tomato diseases. The ANN achieved an accuracy of 85% for tomato. Chen et al. (2020) also trained a deep learning model for plant disease prediction and had an average accuracy of 92.0%, while Wang et al. (2021) developed a robust plant disease detection system that outperformed the mPD-App with an accuracy of 97.62%; the mPD-App had a better precision rate with a score of 95%, as opposed to their precision of 92.81%. Table 2 presents a comparison between mPD-App and some state-of-the-art studies.

**Table 2:** Comparison between mPD-App and some state-of-the-art studies.

**S/N**	**Authors**	**Accuracy**
1	Ma et al. (2018)	93.4%
2	Picon et al. (2019)	87%
3	Durga and Anuradha (2019)	85%
4	Chen et al. (2020)	92.0%
5	Wang et al. (2021)	97.62%
6	Asani et al.—mPD-App	94%

## 5. Conclusion

This study addressed the pressing global challenge of food security, particularly in Sub-Saharan Africa, by developing an image processing system based on convolutional neural network (CNN) for plant disease detection. The mobile-enabled Plant Diagnosis-Application (mPD-App) offers a practical and user-friendly, product-oriented, and accessible system that empowers farmers in Sub-Saharan Africa to detect plant diseases effectively and make informed decisions. The developed CNN-based system leverages deep learning techniques, enabling the computer to autonomously learn relevant features from plant images without extensive human intervention. The use of a robust dataset with a diverse range of plant diseases makes it suitable for crowdsourcing applications among farmers. This research initiative contributes to the field of plant disease detection by developing a user-centric, web-enabled, real-time service that accurately identifies and classifies plant diseases. The mPD-App represents a significant contribution to knowledge as it offers a practical solution to combating plant diseases, empowering farmers to make timely decisions for disease management, reducing crop destruction, and ultimately enhancing food security. The mPD-App can be recommended for deployment in agricultural communities in Sub-Saharan Africa, to improve plant disease detection and management. Future studies could focus on further enhancing the performance and capabilities of the mPD-App. Additional plant disease categories, which is considered as a limitation of this study, may be incorporated into the dataset to make the system more comprehensive. Moreover, continuous updates and improvements can be made to the CNN model to increase accuracy and robustness. Collaboration with local agricultural experts and extension services can also help to gather valuable feedback and insights for refining the system and subsequently developing a crowdsourcing system.

## Data availability statement

The original contributions presented in the study are included in the article/supplementary material, further inquiries can be directed to the corresponding authors.

## Author contributions

Conceptualization and writing—original draft preparation: EA, YO, and AA. Methodology and software: YO. Validation: EA, AA, SV, and JA. Formal analysis and investigation: EK. Resources: AA, SV, and EK. Data curation: AA. Writing—review and editing: SV and JA. Funding acquisition: AA and SV. All authors contributed to the article and approved the submitted version.
