# Corrigendum: mPD-APP: a mobile-enabled plant diseases diagnosis application using convolutional neural network toward the attainment of a food secure world

**DOI:** 10.3389/frai.2023.1325606

**Published:** 2023-11-22

**Authors:** Emmanuel Oluwatobi Asani, Yomi Phineas Osadeyi, Adekanmi A. Adegun, Serestina Viriri, Joyce A. Ayoola, Ebenezer Ayorinde Kolawole

**Affiliations:** ^1^Department of Computer Science, Landmark University, Omu-Aran, Nigeria; ^2^Landmark University SDG 11 (Sustainable Cities and Communities Research Group), Omu-Aran, Nigeria; ^3^School of Mathematics, Statistics and Computer Science, University of Kwazulu-Natal, Durban, South Africa; ^4^Department of Electrical and Computer Engineering, Santa Clara University, Santa Clara, CA, United States; ^5^Department of Agricultural Economics and Extension, Landmark University, Omu-Aran, Nigeria

**Keywords:** mobile-enabled, convolutional neural networks, diseases diagnosis system, SDG2, pathogens

In the published article, there was an error in the email of one of the corresponding authors—Serestina Viriri. Instead of “virirs@ukzn.ac.za”, it should be “viriris@ukzn.ac.za”.

The authors apologize for this error and state that this does not change the scientific conclusions of the article in any way. The original article has been updated.

